# A Unique Presentation of Atypical Hemolytic Uremic Syndrome With Unilateral Blindness and Ischemic Stroke: A Case Report

**DOI:** 10.7759/cureus.73632

**Published:** 2024-11-13

**Authors:** Komal Girdhar, Dharam P Bansal, Puneet Rijhwani, Medha Gupta, Narinder Girdhar

**Affiliations:** 1 Department of General Medicine, Mahatma Gandhi Medical College and Hospital, Jaipur, IND; 2 Department of Internal Medicine, Mahatma Gandhi Medical College and Hospital, Jaipur, IND; 3 Department of Medicine, Dr. Baba Saheb Ambedkar Medical College and Hospital, New Delhi, IND

**Keywords:** adamts 13, atypical hus, malignant hypertention in ahus, plasmapharesis, thrombotic microangiopathy (tma)

## Abstract

This report describes a rare presentation of atypical hemolytic uremic syndrome with multi-system involvement, including unilateral blindness and stroke. Only a few cases of atypical hemolytic uremic syndrome with unilateral blindness as a presentation have been reported and all have been attributed to central retinal artery obstruction (CRAO). This is the first described case to our knowledge of atypical hemolytic uremic syndrome presenting with unilateral blindness, which was caused by grade four hypertensive retinopathy.

## Introduction

Thrombotic microangiopathy (TMAs) is a spectrum of diseases presenting with micro-angiopathic hemolytic anemia, thrombocytopenia, and pathologically having microthrombi, leading to ischemic tissue injury, as defined by Arnold et al. [1]. Thrombotic microangiopathies are seen in diseases like hemolytic uremic syndrome (typical and atypical), thrombotic thrombocytopenic purpura (TTP), autoimmune conditions like systemic lupus erythematosus (SLE), drugs, and pregnancy. Hemolytic uremic syndrome is a thrombotic microangiopathy characterized usually by severe renal impairment along with other end-organ damage and a normal or a slightly reduced ADAMTS13 activity. The most common form (typical hemolytic uremic syndrome) is associated with bloody diarrhea due to Shiga toxin-producing *Escherichia coli*, as also discussed by Mubarik et al. [2]. Atypical hemolytic uremic syndrome is a clinical entity with the triad of hemolysis, consumptive thrombocytopenia, and renal failure. It is often genetic and results from inappropriate activation of the alternative complement pathway, which is a component of the innate immune system. Approximately, 50%-70% of patients with atypical HUS have genetic mutations in complement regulatory genes and a small subset has antibodies against factor F, which is a major regulator of the complement pathway. The most common extra-renal manifestation involves the central nervous system, which could present as marked obtundation, cerebrovascular accidents, seizures, or coma.

## Case presentation

**Figure 1:**
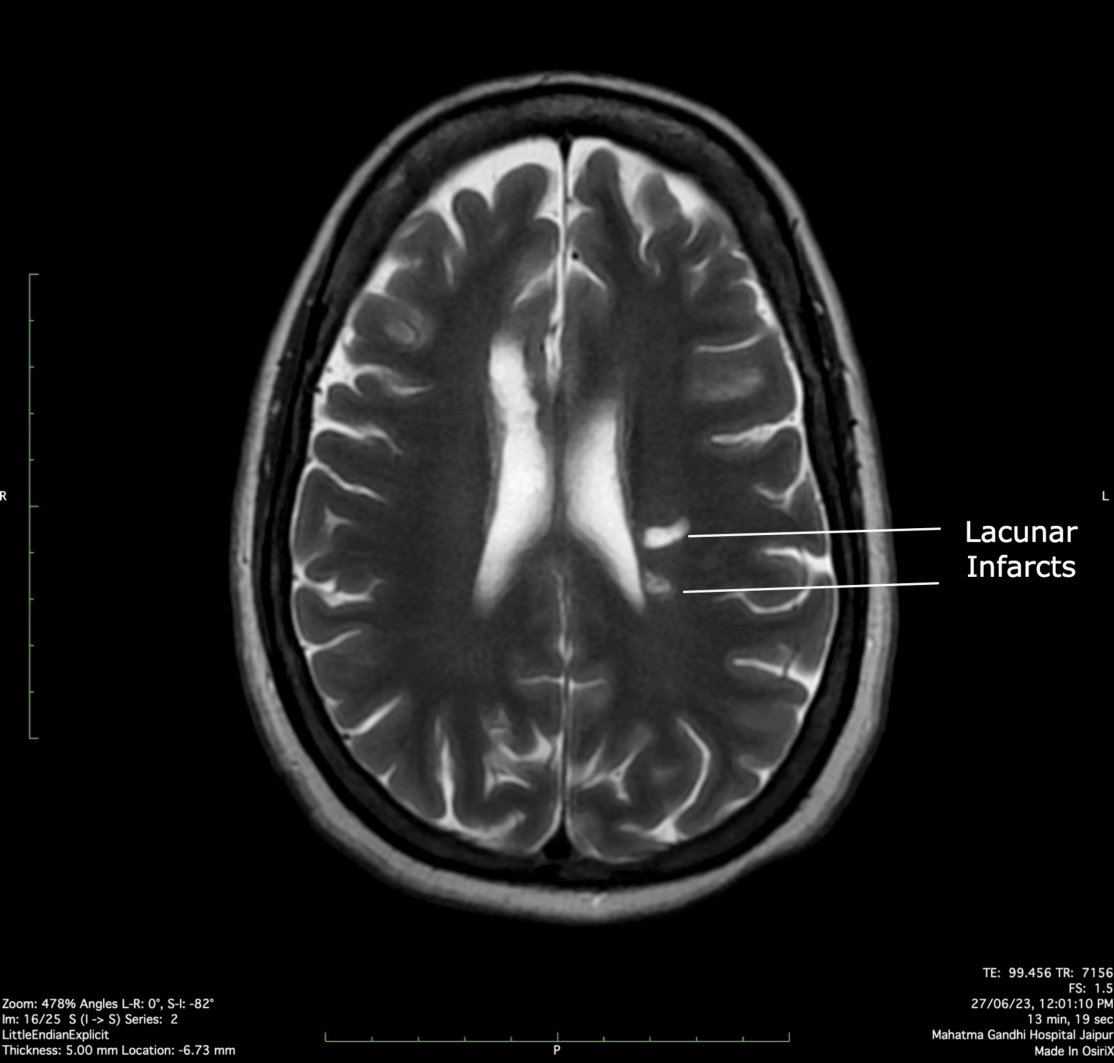
MRI brain suggestive of lacunar infarcts

A 30-year-old male patient who had only a history of progressive diminution of vision in the left eye for two months presented to us with continuous fever recorded at 100-102 degrees Fahrenheit and with chills. It was associated with diarrhea, non-blood or mucous stain, and hematuria for 15 days. The painless hematuria episodes consisted of reddish-colored urine without clots and was eventually followed by oliguria during the intensive care unit (ICU) stay. Upon presentation, his blood pressure was 220/120 mmHg and had pallor and mild icterus, while rest of the general and neurological examination was normal. His labs on admission were hemoglobin level of 10 g% (reference range 11-15 g%), a total leukocyte count (TLC) of 20,000 cells/μL (reference range of 4000-11,000 cells/μL), serum creatinine of 5.3mg/dl (reference range 0.6-1.2 mg/dl), a platelet count of 1,40,000 (reference range 1,50,000 to 4,00,00/μL), an increased serum total bilirubin of 4 mg/dl (reference range 0.3-1.2 mg/dl), indirect bilirubin of 2.6 mg/dl (reference range 0.2-0.8 mg/dl), serum lactate dehydrogenase (LDH) of 540 U/L (reference range 140-280 U/L), and a reticulocyte count of 3.9%(reference range 0.5-2.5%). At this point, we managed him on the lines of bacterial sepsis or tropical infections with acute renal failure and disseminated intravascular coagulation (DIC) with broad-spectrum antibiotics and supportive measures. On day 3 of the hospital stay, he developed frank bleed per rectum with clots, causing a fall of Hb to 5g/dl. The upper gastrointestinal (GI) endoscopy was normal and colonoscopy revealed ileal erosions. His repeat peripheral blood film showed schistocytes and target cells, indicating intravascular hemolysis. Considering a high suspicion of thrombotic microangiopathic anemia (TMAs), he was given fresh frozen plasma (FFP-6 units), which was the only easily available management with us, along with packed red blood cell (RBC) transfusions for anemia. Plasmapheresis was planned. He also developed left-sided hemiparesis with right-sided ptosis and altered sensorium on the fourth day. Magnetic resonance imaging (MRI) of the brain was suggestive of multiple acute and chronic infarcts with chronic micro-bleeds in bilateral basal ganglia regions (Figure 1).

**Figure 2:**
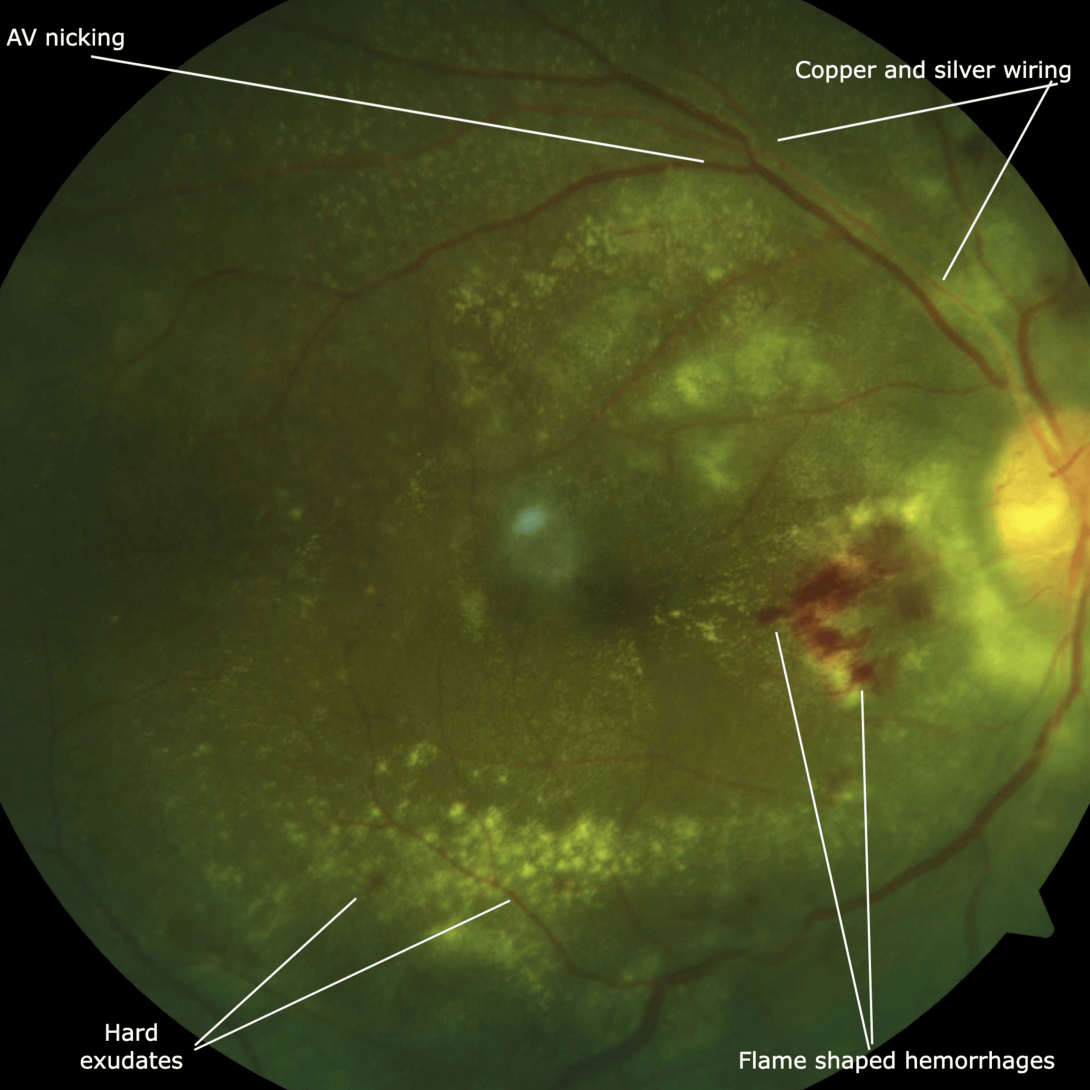
Fundus photography Fundus photography involves photographing the fundus by specialized cameras mainly to see the retina, optic disc, and the macula. Grade 3 hypertensive retinopathy shows copper and silver wiring (thickened arteriolar walls), arterio-venous nicking, soft and hard exudates and flame-shaped hemorrhages.

**Figure 3:**
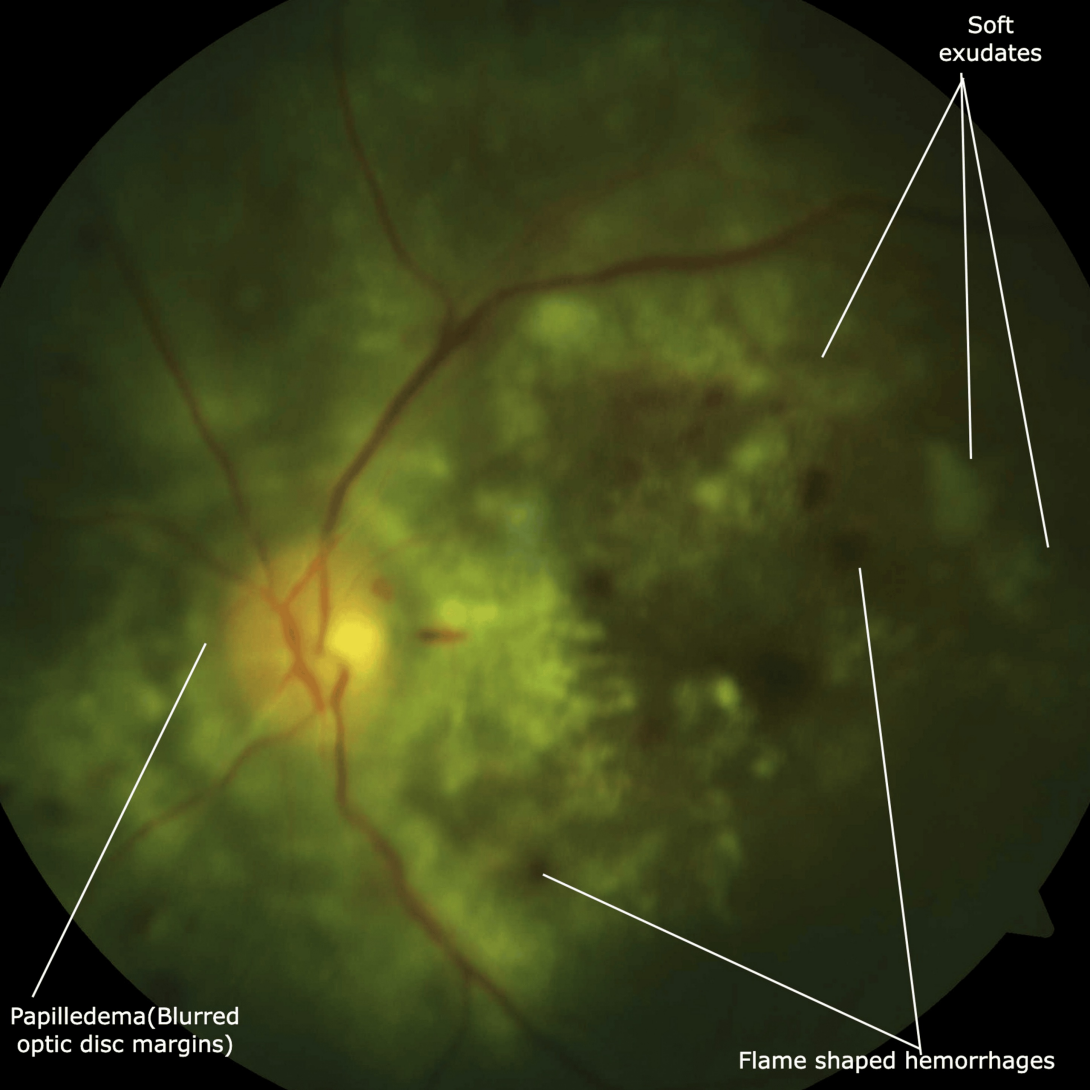
Fundus photography Fundus photography involves photographing the fundus by specialised cameras mainly to see the retina, optic disc and the macula. Grade 4 Hypertensive retinopathy: Post-papilledema changes (blurred optic disc margins), soft exudates and flame-shaped hemorrhages.

In the next three days, he complained of complete vision loss in the left eye. His fundus examination revealed left-sided papilledema (grade 4 hypertensive retinopathy) and grade 1-3 hypertensive retinopathy changes in both eyes (Figures 2, 3).

**Figure 4:**
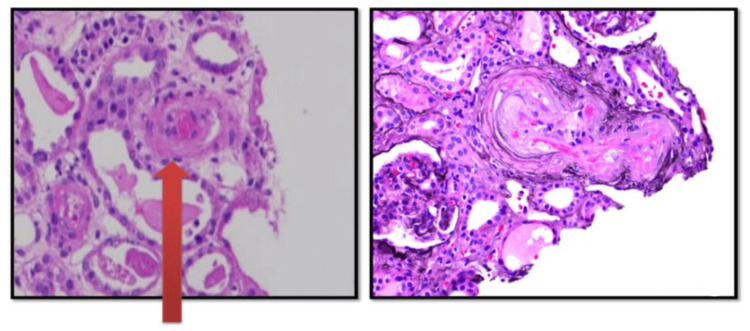
Renal biopsy Biopsy: The pathologist removes the cells and tissues from the body and sees them under the microscope. Hematoxylin and eosin (H&E): Circumferential medial thickening, fibrinoid necrosis and luminal thrombotic occlusion in the arteries.

**Figure 5:**
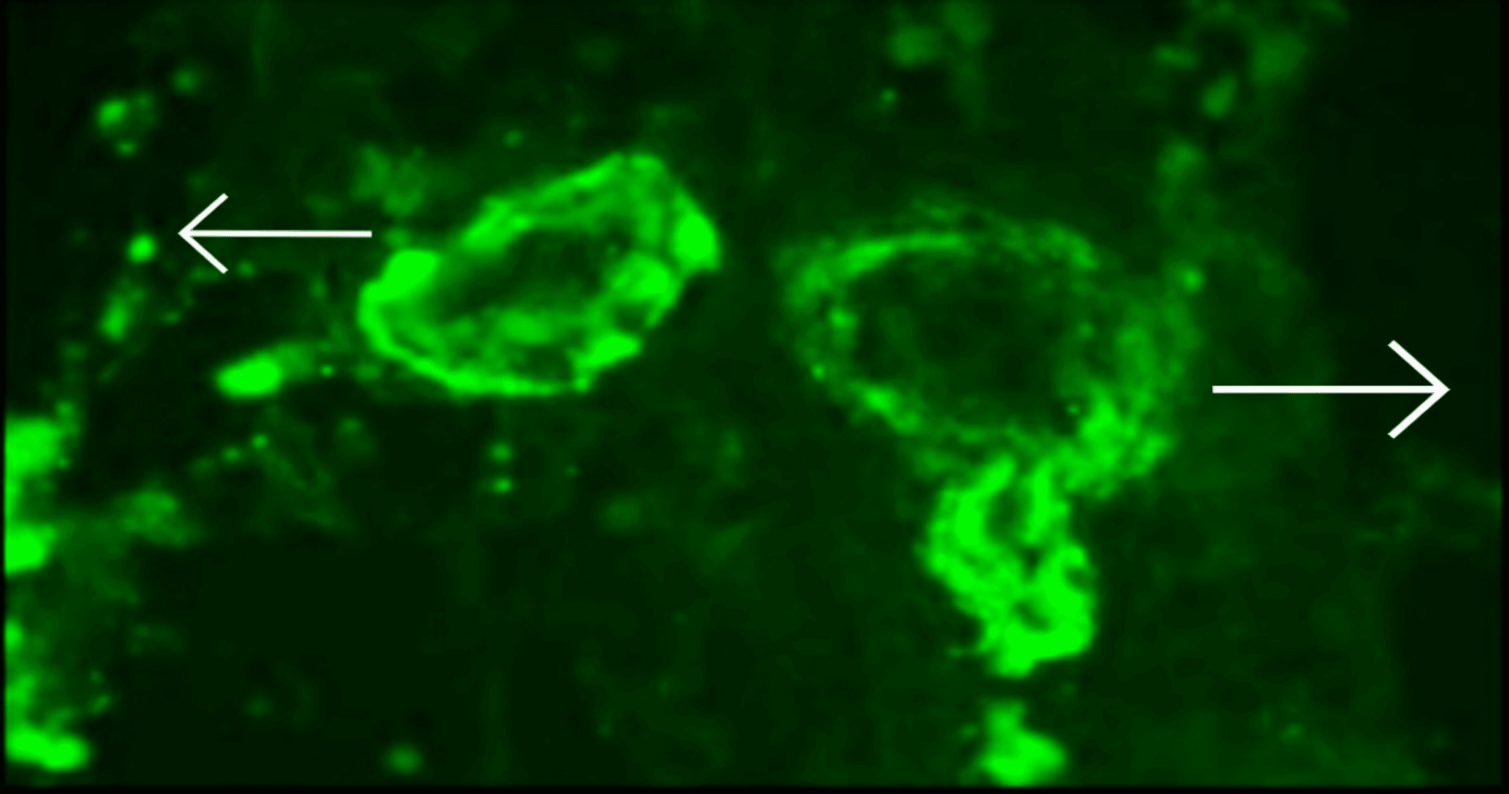
Direct immunoflouresence Direct immunofluorescence (DIF) is a procedure where specific antigens or proteins are detected on renal biopsy specimen with the help of the fluorescence microscope. Direct immunofluorosence on renal biopsy showing Complement (C3) deposits in glomerular capillary membrane.

Homocysteine, anti-nuclear antibodies (ANA) by immunofluorescence assay (IFA), anti-phospholipid antibodies (APLA profile), and anti-neutrophilic cytoplasmic antibodies - cytoplasmic and perinuclear (cANCA, pANCA) were negative. We decided to go for renal biopsy, which on histopathology showed thrombotic microangiopathy (TMA), with glomeruli revealing significant ischemic alterations without significant glomerular immune deposits and severe acute tubular injury with focal chronic interstitial inflammation (Figure 4) but significant C3 deposits in the glomeruli (Figure 5). The serum complement 3 (C3) levels were low, documented as 58.4 mg/dl (normal range 90-180 mg/dl), while the C4 level was 12 mg/dl, which was normal (Figure 4). On the sixth day, he complained of complete loss of vision in the left eye, which was confirmed by no perception of light and no projection of rays in the left eye. Having ruled out central retinal artery occlusion, due to thrombosis of the central retinal artery [3], which is probably the only reported cause of blindness in atypical hemolytic uremic syndrome patients, grade 4 hypertensive retinopathy and papilledema was established as the cause.

The final diagnosis of atypical HUS was considered. There was evidence of hemolysis with microangiopathic hemolytic anemia (MAHA) on renal biopsy with hypocomplementemia (low C3 levels and normal C4 levels) along with extensive renal involvement. Complement H and ADAMTS 13 assay was not performed due to unavailability. These tests are important to make the diagnosis of aHUS since having a significantly low ADAMTS13 (<5%) points to the diagnosis of TTP, while complement H levels are important for the diagnosis of aHUS. Even though we couldn't perform these tests, the rest of the findings pointed to aHUS, as per Carvalho et al. [4]. The patient’s father had died of renal failure and dialysis-related complications at 58 years of age, which indicated a probable positive family history, further strengthening our diagnosis. Before the advent of the C5 inhibitor, plasma transfusions and plasmapheresis were the first-line treatment for aHUS. In our case, we want to suggest that in locations where eculizumab is not available, plasma infusions can be used alongside plasmapheresis.

The patient’s serum creatinine dropped to 2 mg/dl with improvement in weakness. He was finally discharged after a challenging ICU stay of 15 days. He continues to be on our regular outpatient department (OPD) follow-up with stable creatinine levels of 2 mg/dl. As eculizumab remains unavailable in India, this patient was successfully managed by giving plasma transfusions and without hemodialysis.

## Discussion

The diagnosis of aHUS requires recognition of thrombotic microaniopathy: schistocytes, elevated lactate dehydrogenase, decreased haptoglobin, decreased hemoglobin, and thrombocytopenia (platelet count less than 150,000 or > 25% decrease from baseline), as per Nguyen et al. [5]. This should be accompanied by one or more of the following: neurological symptoms, acute renal failure, or gastrointestinal symptoms. Our case represents an unusual presentation of aHUS, which is itself a rare entity. Given the presentation with stroke and unilateral vision loss made it a diagnostic challenge. But keeping a high degree of suspicion, our case was successfully managed in spite of the unavailability of eculizumab in India. Atypical HUS is a disease due to abnormalities in complement pathway activation, which is implicated in destroying microorganisms and damaged cells by enhancing the abilities of phagocytic cells and antibodies, as per Khattab et al. [6]. To our knowledge, this is the first reported case of non-central artery occlusion-related vision loss in atypical HUS. In our patient, there was progressive unilateral vision loss due to grade 4 hypertensive retinopathy secondary to malignant hypertension caused by renal failure. This is in stark contrast to the very few reported cases of central retinal artery occlusion, according to Carvalho et al. [4] and Mehta et al. [3] due to thrombosis of the central retinal artery, which is more acute in onset and also may be reversible after giving eculizumab. In spite of the high specificity of compliment H and ADAM-TS13, we could not perform these analyses due to their non-availability.

## Conclusions

aHUS is a rare disorder with a very poor prognosis. While differentiation between TTP and atypical hemolytic uremic syndrome is difficult, a platelet count of more than 30,000 and a serum creatinine of more than 1.7 to 2.3 makes TTP less likely. Also, since TMAs can involve any organ, except the lungs, a neurological involvement like stroke, even though rare, can still be present in aHUS. A retinal involvement is very rare but of all the reported cases had been attributed to CRAO due TMA in retinal arteries. Blindness due to malignant hypertension in aHUS is the first reported case, to the best of our knowledge. Plasma therapy is still the first-line treatment of such patients where eculizumab is unavailable.
